# A Dynamic Circuit Hypothesis for the Pathogenesis of Blepharospasm

**DOI:** 10.3389/fncom.2017.00011

**Published:** 2017-03-07

**Authors:** David A. Peterson, Terrence J. Sejnowski

**Affiliations:** ^1^Computational Neurobiology Laboratory, Salk Institute for Biological StudiesSan Diego, CA, USA; ^2^Institute for Neural Computation, University of California, San DiegoSan Diego, CA, USA

**Keywords:** striatum, dopamine, blepharospasm, dystonia, rodent models

## Abstract

Blepharospasm (sometimes called “benign essential blepharospasm,” BEB) is one of the most common focal dystonias. It involves involuntary eyelid spasms, eye closure, and increased blinking. Despite the success of botulinum toxin injections and, in some cases, pharmacologic or surgical interventions, BEB treatments are not completely efficacious and only symptomatic. We could develop principled strategies for preventing and reversing the disease if we knew the pathogenesis of primary BEB. The objective of this study was to develop a conceptual framework and dynamic circuit hypothesis for the pathogenesis of BEB. The framework extends our overarching theory for the multifactorial pathogenesis of focal dystonias (Peterson et al., 2010) to incorporate a two-hit rodent model specifically of BEB (Schicatano et al., 1997). We incorporate in the framework three features critical to cranial motor control: (1) the joint influence of motor cortical regions and direct descending projections from one of the basal ganglia output nuclei, the substantia nigra pars reticulata, on brainstem motor nuclei, (2) nested loops composed of the trigeminal blink reflex arc and the long sensorimotor loop from trigeminal nucleus through thalamus to somatosensory cortex back through basal ganglia to the same brainstem nuclei modulating the reflex arc, and (3) abnormalities in the basal ganglia dopamine system that provide a sensorimotor learning substrate which, when combined with patterns of increased blinking, leads to abnormal sensorimotor mappings manifest as BEB. The framework explains experimental data on the trigeminal reflex blink excitability (TRBE) from Schicatano et al. and makes predictions that can be tested in new experimental animal models based on emerging genetics in dystonia, including the recently characterized striatal-specific D1R dopamine transduction alterations caused by the GNAL mutation. More broadly, the model will provide a guide for future efforts to mechanistically link multiple factors in the pathogenesis of BEB and facilitate simulations of how exogenous manipulations of the pathogenic factors could ultimately be used to prevent and reverse the disorder.

## Introduction

The ability to keep your eyes open is an obvious prerequisite for vision, but even this simple function is dramatically impaired in blepharospasm (“benign essential blepharospasm,” BEB). BEB is characterized by involuntary eye closures that impair many activities of daily living, including driving, reading, and walking. There is visual impairment in an estimated 50–70% of patients, and in severe cases BEB causes functional blindness. The pathophysiology of BEB is not well understood. Possible environmental factors include keratitis, dry eyes, greater sunlight exposure, and blepharitis (Digre, 2015). Many patients complain of photophobia. BEB remits in less than 1% of patients, and leads to significant social disability and decreased quality of life. Many oral medications have been tried, but they are minimally effective (Eftekhari et al., 2015). Some medications have undesirable side effects, producing Parkinsonism or blurred vision. Botulinum neurotoxin (BoNT) injections have become the mainstay treatment for BEB. But the injections are costly and must be repeated every 3–4 months in perpetuity. In some cases they also produce transient adverse side effects, including ptosis and diplopia. Over 70% of patients report difficulties driving and reading 2 or more weeks before the next injection, and over time some patients develop resistance to the toxin (Esposito et al., 2014). Patients refractory to BoNT have surgical options, including deep brain stimulation (DBS) or myectomies. But the myectomies for BEB are among the most challenging procedures in oculoplastic surgery. For some surgical patients, the procedure does not necessarily supplant the need for continued BoNT injections. As a result of incomplete treatment efficacy, for many patients BEB leads to a lifetime of chronic disability, occupational failure, social withdrawal, and depression. We could develop principled strategies for preventing and reversing the disease if we knew the pathogenesis of BEB.

In this paper, we develop a conceptual framework and dynamic circuit hypothesis for the pathogenesis of BEB. Section Conceptual Framework lays out the conceptual framework based on concepts of sensorimotor plasticity and “2-hit” models for the pathogenesis of dystonia, and BEB in particular. In Section Dynamic Circuit Hypothesis for the Pathogenesis of BEB we build on the conceptual framework to formulate a basal ganglia-based circuit hypothesis that explains Schicatano et al.'s 2-hit rodent model of BEB, including the temporal dynamics of the pathophysiology at two time scales. In Section Computational Model Design Considerations we discuss strategies for developing computational models based on the hypothesis. In Section Broader Significance we conclude with the significance of our framework for broader research efforts to improve treatments for BEB and other focal dystonias.

## Conceptual framework

### Sensorimotor plasticity and the hyperexcitable blink reflex

BEB is associated with enhanced plasticity in the overall circuits mediating blink sensorimotor function. A broad body of research into BEB suggests that the disease involves disordered sensorimotor integration (Feiwell et al., 1999; Berardelli and Curra, 2002). One of the most consistent specific findings is hyperexcitability in the blink reflex (Hallett, 2002). This is most notable in the late component of the blink reflex, measured with rectified electromyography of the orbicularis oculi (OO) muscles in response to stimulation of the supraorbital branch (SO) of the trigeminal nerve (Berardelli et al., 1985). The hyperexcitability has been attributed to abnormalities in sensorimotor brainstem loops (Gómez-Wong et al., 1998; Gong et al., 2003), possibly involving some form of positive feedback (Nguyen and Kleinfeld, 2005). These circuits exhibit remarkable plasticity, which in the non-pathological case provides an adaptive mechanism for coping with ophthalmic challenges such as dry eye by increasing blink rates to maintain corneal health. This plasticity can be experimentally manipulated with high-frequency stimulation of the trigeminal nerve temporally coordinated with reflex blinks (Mao and Evinger, 2001). Interestingly, this form of experimentally-induced plasticity of the overall blink reflex circuit is enhanced in BEB patients (Quartarone et al., 2006a). The notion of abnormal, maladaptive plasticity is gaining broader interest in the dystonia community (Berardelli et al., 1998; Hallett, 1998, 2001, 2006; Sanger and Merzenich, 2000; Altenmüller, 2003; Quartarone et al., 2006b, 2009; Rosenkranz et al., 2007; Torres-Russotto and Perlmutter, 2008), including in BEB research.

### The “2-hit” model for dystonia

Several investigators (Topp and Byl, 1999; Sanger and Merzenich, 2000; Hallett, 2002; Quartarone et al., 2006b, 2008; Torres-Russotto and Perlmutter, 2008; Peterson et al., 2010) have suggested that two factors jointly underlie the pathophysiology of dystonia: “environmental” factors like peripheral injury or repetitive use and subtly abnormal biological substrates of plasticity. That both factors may be necessary is consistent with the findings of reduced penetrance in the genetic contributions to most forms of dystonia, and only limited data thus far for a genetic contribution in BEB (Misbahuddin et al., 2002; Defazio et al., 2003, 2007; Clarimon et al., 2007). As a specific instance of the “2-hit” theme, we previously postulated that a combination of abnormal patterns of sensorimotor state space utilization and abnormal dopaminergic signaling in the striatum could induce pathological reinforcement learning that leads to dystonia (Peterson et al., 2010). Evidence specific to BEB, as laid out in the next section, suggests that this applies to BEB.

### A version of the “2-hit” model for BEB

Some of the strongest evidence to date for this “two-factor” concept implicates ophthalmic and dopaminergic factors in the development of BEB (Evinger, 2013, 2015). BEB is frequently associated with ophthalmic challenges, such as irritation, injury, dry eye, or other diseases of the anterior segment of the eye (Elston et al., 1988; Pita-Salorio and Quintana-Conte, 1988; Hallett, 2002; Martino et al., 2005). Dry eye increases blink oscillations (Evinger et al., 2002), and experimentally-produced lesions that reduce eyelid motility induce a mild dry eye (Evinger, 2005) and increase blink reflex excitability (Schicatano et al., 1997, 2002). The most common interpretation is that these ophthalmic challenges induce an adaptive increase in OO drive to compensate for lid weakness. This adaptive process is a natural means of maintaining proper function, but may become maladaptive in BEB (Evinger and Manning, 1988). Interestingly, even a transient trigger can lead to a persistent problem, because lid closure spasms persisted even after full recovery of facial nerve function (Evinger, 2005).

Dopamine (DA) also likely plays a subtle but significant role in BEB. The dopamine system is implicated as a potential factor in many different forms of dystonia (Augood et al., 1999, 2004; Perlmutter and Mink, 2004; Breakefield et al., 2008; Wichmann, 2008; Peterson et al., 2010). In tardive dystonia (e.g., due to neuroleptics that block DA receptors), the initial presentation is commonly BEB. Decreased DA increases blink durations and the trigeminal reflex blink excitability (TRBE; Basso et al., 1993; Esteban, 1999; Peshori et al., 2001). Eye disease increases dramatically with age, especially in 40–60 year olds, a period coincident with decreased production of DA in the SNc and coincident with the typical age of BEB onset (Martino et al., 2005). BEB symptoms respond to apomorphine, a non-selective DA agonist (Cattaneo et al., 2005). BEB has been associated with polymorphisms in the gene that codes for the D5 dopamine receptor (Misbahuddin et al., 2002). Dopamine mediates nicotine's influence on the blink reflex (Evinger et al., 1993), and there is decreased binding of the D2-family of striatal DA receptors in BEB (Perlmutter et al., 1997; Horie et al., 2009).

### A rodent model of BEB

In a rat model of BEB, Schicatano et al. (1997) demonstrated that the joint contribution of ophthalmic and dopaminergic factors could be critical in the pathogenesis of the disorder. Specifically, they found that only the *combination* of dopaminergic and ophthalmic factors dramatically increased blink reflex excitability and recapitulated the symptoms of BEB. They combined a weakening of the orbicularis oculi muscles that increased spontaneous blink rate with a subclinical 6-OHDA lesion of the dopamine system in the basal ganglia. They tested the animal's trigeminal reflex blink excitability (TRBE). The blink reflex is commonly quantified with rectified EMG of the orbicularis oculi (OO) muscles. The first burst of EMG, called the “R1,” precedes the blink and is ipsilateral to the stimulus. The second burst of EMG, called the “R2,” coincides with the blink and is bilateral. The TRBE is measured by the relative change in the magnitude of the R2 component of the blink reflex to a “test” SO stimulus at least 50 ms after an equivalent conditioning stimulus.

(1) $$\begin{array}{l} TRBE=\;\frac{\int R2_{test}}{\int R2_{condition}} \end{array}$$

**Figure 1:**
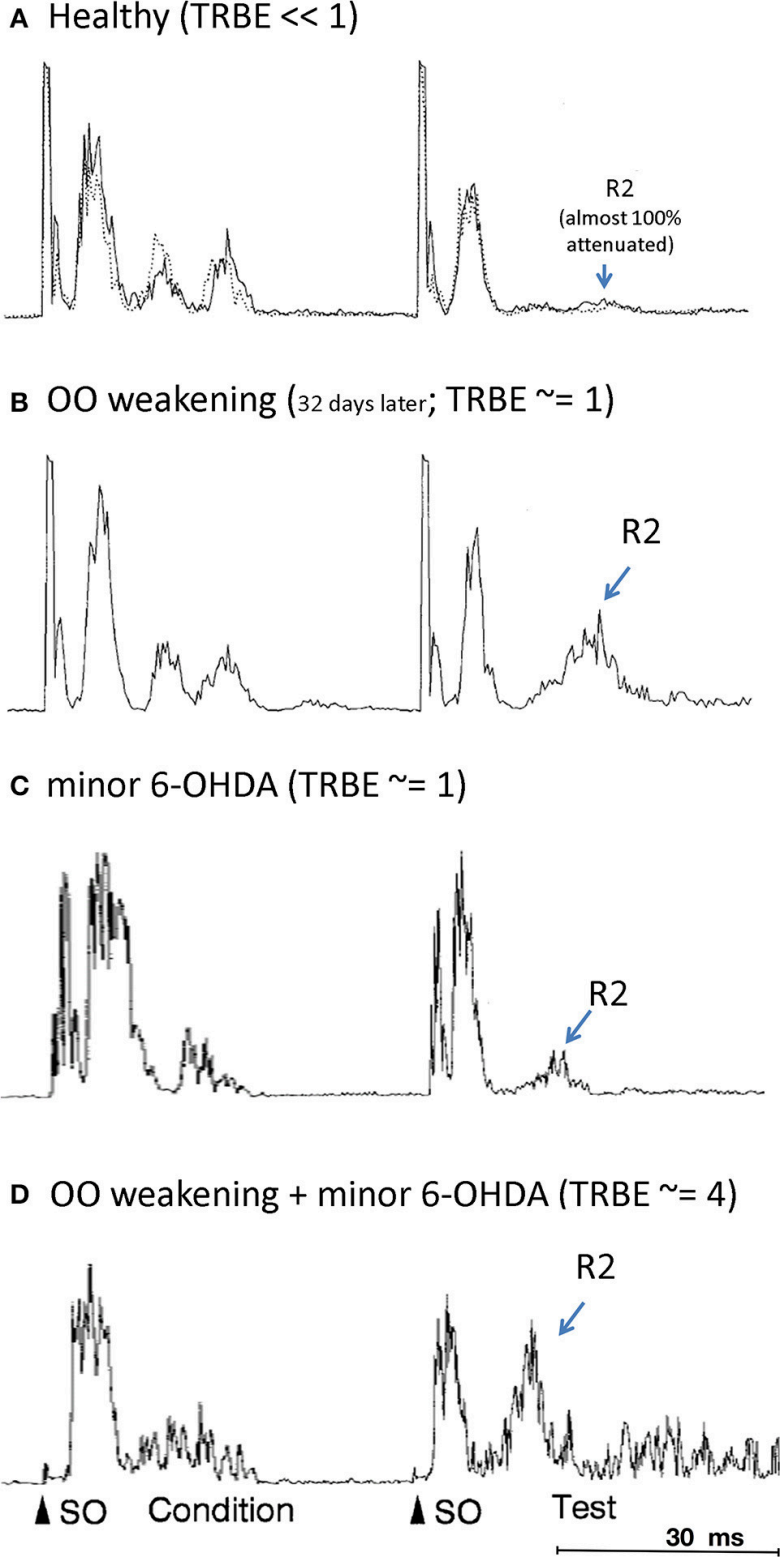
**Orbicularis oculi EMG responses to paired pulse stimulations (“condition”, “test”) to the supraorbital branch (SO) of the trigeminal nerve**. The R2 response component is labeled only for the test stimulus. (OO EMG is rectified, amplitude given in arbitrary units; all adapted from Schicatano et al., 1997). **(A)** Healthy animal, prior to OO and 6-OHDA lesions (from Schicatano Figure 2A top panel; solid and dotted responses are on separate, sequential days). **(B)** After OO weakening (from Schicatano Figure 2A bottom panel). **(C)** After minor 6-OHDA lesion (from Schicatano Figure 3A, temporally scaled to approximate stimulus timing in **A,B**). **(D)** After minor 6-OHDA lesion and approx. 20 days after OO weakening (from Schicatano Figure 3B, temporally scaled as in **C**). Note lid closure spasms (LCS) after test R2.

The effects on the physiological measures of TRBE are depicted in Figure 1 and summarized in Table 1. Only the animals with both factors manipulated exhibited an approximately 4-fold increase in the TRBE. They also exhibited spontaneous lid closure spasms, mimicking the symptoms of BEB in humans. Neither of the individual manipulations in isolation created the BEB phenotype.

**Table 1:** **scenario-specific OO physiology**.

	**TRBE**	**Lid closure spasms (LCS)**	
	**(R2 test /R2 cond)**	**Reflex ?**	**Spontaneous ?**
Healthy	<1	–	–
Weakened OO	1	–	–
6-OHDA	1	–	–
6-OHDA + weakened OO	4	Yes	Yes

## Dynamic circuit hypothesis for the pathogenesis of BEB

### The hypothesis

The experiments by Schicatano et al. opened a door into a mechanistic model of BEB that had some phenotypic validity relative to the human condition. However, the study also raised questions that remain unanswered to this day. They suggested that the dopamine deficit in the basal ganglia created a “permissive condition” by reducing the inhibition on the trigeminal reflex circuit. However, it remains a mystery *how* the ophthalmic and dopaminergic factors could *interact* to produce the increased TRBE and the expression of BEB. For example, at the short time scale, it is not clear why this disinhibition would be selective for the R2 response to only the test stimulus, and not the preceding conditioning stimulus.

We hypothesize that the propagation delays associated with the long loop—from brainstem to thalamus_VPN to somatosensory cortex to striatum to SNr back to brainstem—in combination with an altered input/output weight matrix in the striatum, selectively disinhibits the R2 response in the TRBE. Furthermore, at the long time scale, there were multi-day delays between the DA and ophthalmic interventions and when the TRBE was tested. Presumably these were to enable the animals to recover from surgeries. However we hypothesize that those multi-day delays also allow for slower circuit plasticity to reshape the weight matrix central to the action selection function of the striatum, such that instantaneous basal ganglia outflow to the blink circuitry in the brainstem becomes contingent upon recent periocular activity in a pathological fashion.

### A BG role in BEB

#### Brain regions implicated in BEB

A wide array of evidence implicates regions outside of the brainstem in BEB. BEB is associated with abnormal gray matter volume (Etgen et al., 2006; Obermann et al., 2007; Martino et al., 2011) and glucose metabolism (Eidelberg et al., 1998; Hutchinson et al., 2000) in the cerebellum, the basal ganglia, and the cortex. Many cortical areas, virtually all of which have projections to the basal ganglia, are involved in facial muscle control (Morecraft et al., 2001, 2004; Sohn et al., 2004; Gong et al., 2005) including blinking (Baker et al., 2003; Evinger and Perlmutter, 2003). Some set of “higher” brain regions are also implicated in modulating the blink reflex, because stimulus anticipation influences the blink reflex (Ison et al., 1990).

#### BEB and the BG

Strokes involving the striatum can produce BEB (Grandas et al., 2004). In BEB patients, striatal volumes are abnormal (Etgen et al., 2006; Obermann et al., 2007) and metabolism is higher than normal (Esmaeli-Gutstein et al., 1999). BEB patients also exhibited differential recruitment of specific subregions of striatum during lid closing spasms compared to control subjects engaged in frequent blinking (Schmidt et al., 2003). In contrast, the frontal cortex and cerebellum did not exhibit differential recruitment in the two groups. Likewise, a manual sensorimotor control task induced greater activation in BEB patients than in controls in the basal ganglia but not in the cortex (Obermann et al., 2008). A basal ganglia abnormality in BEB would also be consistent with the higher incidence of OCD symptoms in BEB than in hemifacial spasm (Broocks et al., 1998) and the higher incidence of OCD in primary focal dystonias overall compared to the general public (Cavallaro et al., 2002). Finally, deep brain stimulation of the globus pallidus internal segment (GPi), one of the primary output structures of the basal ganglia, also reduces blink reflex excitability (Tisch et al., 2006).

#### BG outflow and the blink reflex

**Figure 2:**
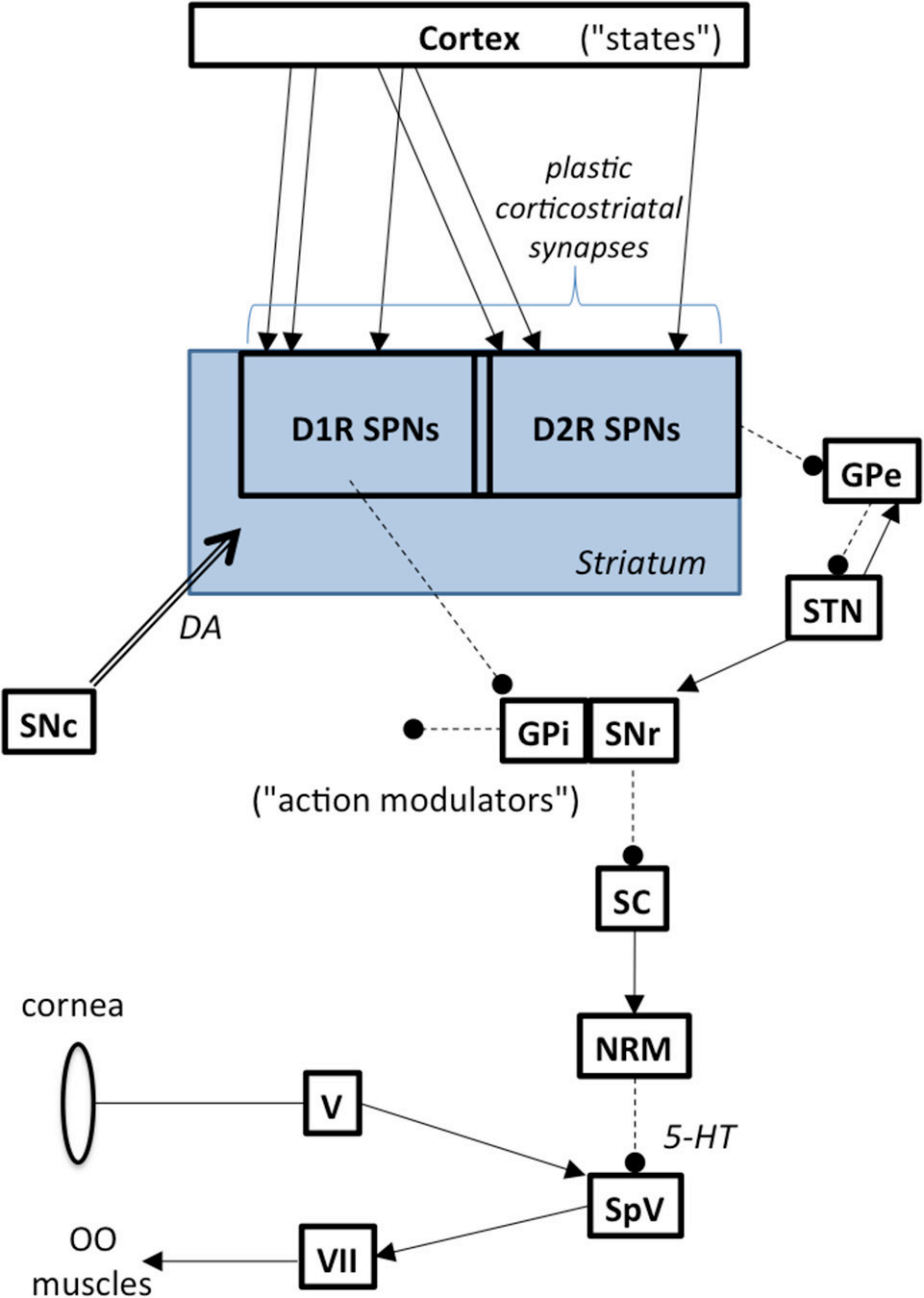
**Basal ganglia to brainstem pathways influencing reflex blinks**. Abbreviations: GPe, globus pallidus external segment; GPi, globus pallidus internal segment; NRM, nucleus raphe magnus; OO, orbicularis oculi; SC, superior colliculus; SNc, substantia nigra pars compacta; SNr, substantia nigra pars reticulata; SPN, spiny projection neurons; SpV, spinal trigeminal nucleus; STN, subthalamic nucleus; V, trigeminal ganglion, VII, facial motor nucleus.

The basal ganglia modulate the blink reflex (Berardelli et al., 1985; Evinger and Manning, 1993). The GPi is the primary output nucleus in the basal ganglia and it is commonly associated with somatotopic musculoskeletal motor function. The substantia nigra pars reticulata (SNr), is another key output nucleus in the basal ganglia. The SNr has descending outputs to, among other targets, the superior colliculus (SC). This pathway modulates peri-ocular motor function. Although the exact nature of how the BG influence the blink reflex remains uncertain, Basso (Basso and Evinger, 1996) postulated the sequential chain depicted in Figure 2, namely that increased SNr activity leads to increased inhibition of SC, leading to decreased excitation of the nucleus raphe magnus and decreased inhibition of trigeminal blink reflex circuits. This is consistent with evidence showing that SC stimulation suppresses the blink reflex (Gnadt et al., 1997) and that the SC is hypometabolic on FDG-PET in BEB (Emoto et al., 2010). Indeed, the pathway from SNr to the trigeminal nucleus has long been suspected to be involved in BEB (Hotson and Boman, 1991).

#### Nested loops

**Figure 3:**
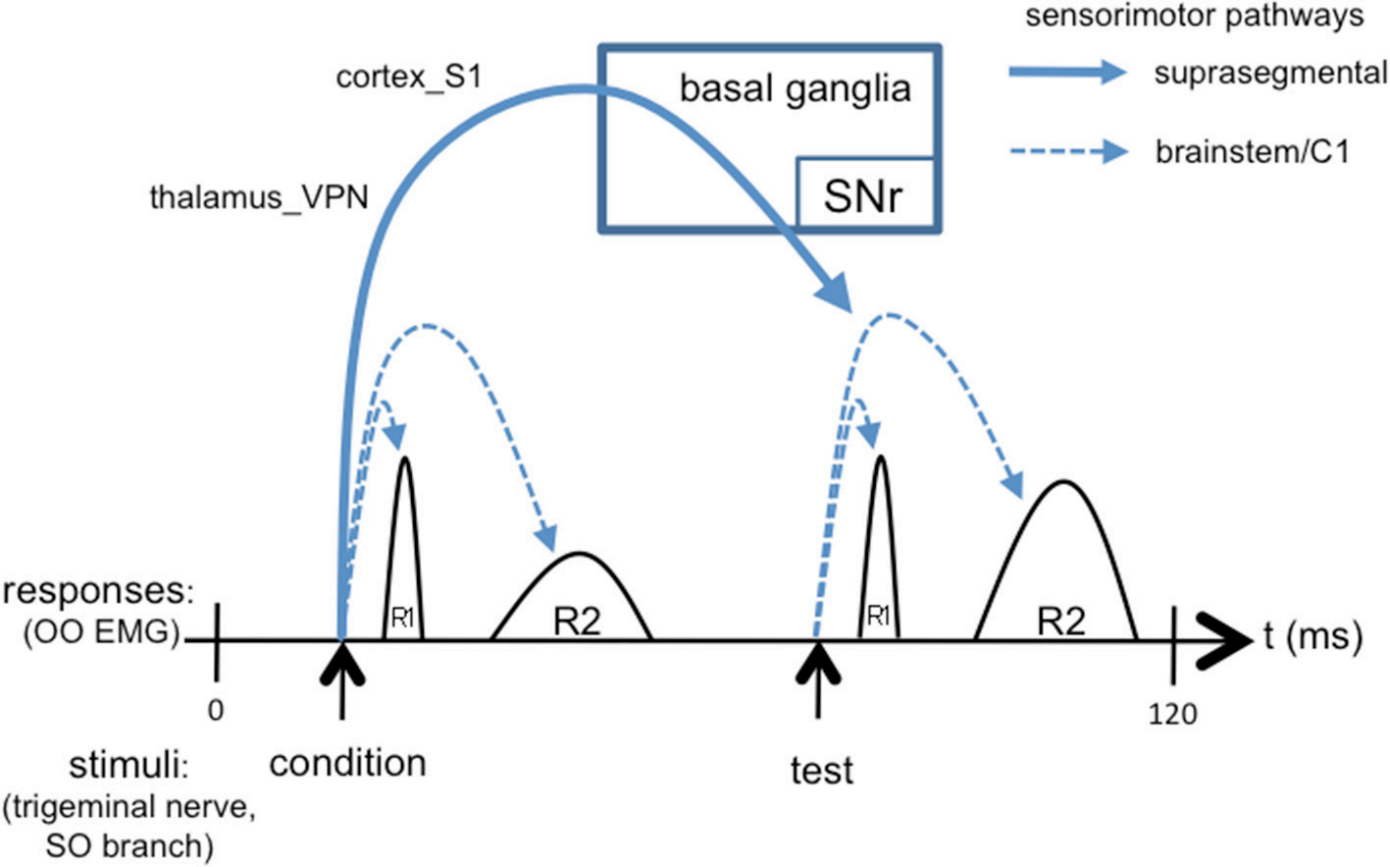
**Simplified schematic of the TRBE, overlaid with signaling cascades including not only short loops in brainstem but also long loops through suprasegmental structures**. The conditioning stimulus evokes suprasegmental responses through thalamus, cortex, and basal ganglia output pathways back to trigeminal reflex circuits in brainstem [stimulus artifact omitted].

We suggest that the ascending trigemino-thalamocortical, and then corticobasal ganglionic, pathways, may mediate the differential response to the second (“test”) versus the first (“condition”) stimuli that underlies the TRBE, as depicted in Figure 3. This is consistent with the typically 50 ms or greater ISIs used in paired pulse paradigms, given that somatosensory evoked potentials are detectable in the cortex with approx. 20 ms latency. The R2 component is influenced by descending projections from cortex and the basal ganglia (Esteban, 1999), likely involving sensory pathways including thalamus_VPN and the S1 cortical area, as evidenced by stroke cases (Kimura et al., 1985; Chia, 1997; Spissu et al., 2004). The basal ganglia-thalamocortical loops have been suggested to modulate the TRBE (Tisch et al., 2006) and to be involved in BEB (Colosimo et al., 2010).

### Action selection learning

#### Dopamine function in the basal ganglia is abnormal in BEB

There is decreased binding of the D2-family of DA receptors in striatum in cranial dystonia, including BEB (Perlmutter et al., 1997; Horie et al., 2009). Basal ganglia dopamine also mediates nicotine's influence on the blink reflex (Evinger and Manning, 1993; Evinger et al., 1993).

#### Dopamine function in the basal ganglia mediates sensorimotor learning

The classic clinical models of the basal ganglia in movement disorders highlight the *static* role of dopamine (Gale et al., 2008). For example, dopamine levels influence the relative activation of the “go” D1-family receptor dependent pathway and the “no go” D2-family receptor dependent pathways in the hypokinetic vs. hyperkinetic features of movement disorders. In another model, these pathways are combined with the divergent excitation from the subthalamic nucleus (STN) to create surround inhibition that improves the relative selectivity attributed to specific action plans. However, the *dynamic* influence of dopamine in the basal ganglia is an underappreciated factor in dystonia research (Utter and Basso, 2008), and may play a critical role in the pathogenesis of the disease. In fact, some investigators have suggested that the classic view of the basal ganglia role in motor modulation may in fact be secondary to a more general purpose role in learning (Wickens, 2009). Striatal synaptic plasticity plays an important role in sensorimotor learning (Graybiel et al., 2000; Pisani et al., 2005; Yin and Knowlton, 2006; Kreitzer and Malenka, 2008; Horvitz, 2009). Much of that research has implicated plasticity specifically in corticostriatal synapses (Kreitzer and Malenka, 2008). The striatum is a dominant recipient of projections from SNc DA cells; DA markers in the striatum are among the densest in the nervous system (Lavoie et al., 1989). Corticostriatal potentiation is correlated with learning rate in an intracranial self-stimulation paradigm involving stimulation of SNc DA neurons (Reynolds et al., 2001) and this was interpreted as a cellular instantiation of sensorimotor learning, i.e., how context-sensitive motor behavior is shaped (Reynolds et al., 2001; Wickens et al., 2003).

#### The basal ganglia likely play a critical role in mediating the interaction of the ophthalmic and dopaminergic factors in BEB

The plasticity of the corticostriatal synapse is a function of not only dopaminergic afferents from SNc but also inputs from cortex and the output of the striatal projection cells, the spiny projection neurons (SPNs). Because the striatum receives convergent input from most of the cerebral cortex, it is positioned to integrate broadly defined sensory and motor plan information. By virtue of its direct and indirect projections to basal ganglia outputs, the striatum also influences how the basal ganglia modulate transitions to future “actions” (Yin, 2016). Behavior involves sampling the space of “state-to-action” mappings. In this context, “state” refers to the instantaneous combination of sensory representations and possible motor plans encoded in cortex. “Action” refers to the “next action” biases determined by BG output. “Mappings” refers to how action biases are formulated from states. The state-to-action mapping is used in a continuous fashion iterated over time. Importantly, the mapping is also potentially updated in a continuous fashion and updates depend in part on which state/action combinations are used. In this framework, the brainstem and associated cerebellar circuits that mediate the adaptive response to peripheral ophthalmic challenges induce a shift in how the “state-to-action” space is sampled, or “used.” In the simplest example, dry eye can evoke increased blinking, whereby the associated sensory-motor “space” is sampled more frequently. Indeed, repeated use is a requisite for many forms of learning, particularly the forms of procedural learning for which the basal ganglia has long been implicated. In summary, specific forms of “use” and dopamine function conspire in the basal ganglia to influence sensorimotor map formation.

## Computational model design considerations

Based on our circuit hypothesis, we outline in this section suggestions for designing corresponding computational models. An important aspect will be to model network-level plasticity in the basal ganglia to simulate how the two factors lead to the hyperexcitable blink reflex in BEB. We suggest development of a computational model of dopamine-mediated plasticity in the basal ganglia network that can be tested with the data from Schicatano's animal model of BEB (1997). A neuronal network model of the cortico-basal ganglia circuits mapping sensory and motor plan states to motor outputs is depicted in Figure 2.

### Model architecture

The model network includes cortex encoding generalized state information (including somatosensory and motor plan), the conjunction of direct and indirect striatal-pallidal pathways as mappings from “states” to “action modulations” in SNr, and dopaminergic input from the substantia nigra pars compacta (SNc). Several components of the basal ganglia network are omitted for simplicity, including striosomes, their projections to SNc, and projections from cortex to STN.

Beyond a general cortical representation of state information, it may be worth including primary motor cortex (M1) as a separate node. It is a point of convergence for cortico-cortical, cerebello-thalamocortical, and BG-thalamocortical networks. It has a key role in other long-loop reflexes, such as the long-latency stretch reflex implicated in skeletal muscle spasticity, and pathophysiology in M1 may be sufficient to cause some features of dystonia as illustrated by M1 simulations accelerated with customized hardware (Sohn et al., 2015). However, for forms of dystonia where altered dopamine signaling is implicated, we need a better understanding of the relative importance of striatal versus cortical dopamine-mediated plasticity in modifying action selection. Regardless of the influence of dopamine in cortex, the exquisitely timed overlay of motor cortex and SNr/brainstem projections to the trigeminal nucleus will need to be elucidated to fully understand how the blink reflex excitability is modulated in BEB.

Historically a majority of theoretical and computational models of dopamine's role in BG circuitry have omitted or downplayed the STN and GPe. Yet there is mounting evidence for their critical role in BG physiology and they do receive dopaminergic innervation from SNc. However, the role of dopamine receptors in their physiology is relatively understudied compared to the striatum, and we are unaware of literature on how dopamine modulates synaptic plasticity in the STN and GPe. Furthermore, it is not known whether the D2 dopamine receptor binding in focal dystonias that is altered in striatum is also altered in STN and GPe. Neverthless, altered dopamine signaling in these nuclei could contribute to BEB pathophysiology by way of the SNr and the SNr's brainstem targets in at least two non-mutually exclusive ways. First, altered activity in the divergent glutamatergic projection from STN to SNr could induce a global excitation of SNr that would disinhibit downstream targets in brainstem. Indeed deep brain stimulation of STN for Parkinson's disease (PD) can alleviate (or, paradoxically for some patients, induce) apraxia of lid opening, a symptom common in isolated blepharospam (Weiss et al., 2010). A clearer understanding of the mechanisms awaits a clearer understanding of the mechanism of action of DBS itself, as the relative contribution of activation of cell bodies and fibers of passage remains unclear. Second, there is evidence that the altered dopamine signaling of Parkinsonism leads to pathological oscillations in the STN-GPe loop. As a result, there would be a disruption in the normal temporal patterns of STN output to SNr, again cascading to altered signaling in the brainstem targets downstream of SNr. With the evolution of synergistic experimental and theoretical work on that basal ganglia subcircuit and DBS mechanisms of action, they should be incorporated into models of the broader basal ganglia network.

### Model neurons

A network model will need to be sufficiently computationally efficient to be able to simulate the effect of dopamine-mediated plasticity of corticostriatal synapses over protracted periods of sensorimotor “use.” Thus the model should use biologically realistic but computationally efficient spiking neurons, as for example the variants on the Izhikevich neuron (Izhikevich, 2003) used by Gurney et al. (2015).

We advocate use of a model that incorporates known information about both the overall BG circuitry and dopamine-dependent synaptic plasticity rules. To our knowledge, Gurney et al. (2015) is the most rigorous and representative computational model incorporating these features available to date. However, Gurney et al. used rate-based neurons to represent a few channels in each of the BG nuclei outside of the striatum. To ultimately facilitate linking the model to experimental data on oscillations in the overall BG network, which have been implicated in movement disorders pathophysiology (Schroll and Hamker, 2016), one should use spiking neurons throughout the model architecture. This direction will be facilitated not only by continued advances in computational resources, but also by expanded knowledge about how best to parameterize features of dopamine-modulated synaptic plasticity at synapses onto the other classes of neurons in the BG beyond SPNs in the striatum. Thus, we advocate a staged approach, involving in the first phase an approach analogous to that of Gurney et al., and in a subsequent phase pending more complete experimental data on DA-mediated synpatic plasticity, extending the use of spiking model neurons to all of the other nodes in the network.

### Model synaptic learning rules

Use of spiking neurons will allow the model to take advantage of spike-timing dependent plasticity (STDP) rules for modifying the strength of the glutamatergic input weights at the cortico-striatal synapses on to spiny project neurons. Although such models have been based on dopamine-dependent STDP rules specific to D1- and D2-type dopamine receptor-containing SPNs (Shen et al., 2008), there remains debate about how those *in vitro* results, and manipulations of inhibition in the slice preparation, map to the *in vivo* setting. Future advances in elucidating those STDP rules for the corticostriatal synapse should be closely monitored and incorporated into future models of striatal network plasticity. One possibility is that a unified formulation based on cytoplasmic calcium dynamics may emerge. A calcium-based formulation for synaptic plasticity may resolve debates about STDP rules and facilitate combining short- and long-term plasticity. Though initially complex, such a formulation could, in principle, be implemented with computationally efficient approximations.

### Model inputs

For inputs, the ophthalmologic factor can be represented as either modified somatosensory input from the trigeminal system, or as increased blinking, or both. The OO muscle weakening is suggested to be about “30%” (Evinger, 2005), consistent with the fact that the OO muscle is innervated by the zygomatic and temporal branches of facial nerve (Ouattara et al., 2004) yet only the zygomatic branch is sectioned. As a result, the magnitude of the eyelid proprioceptive signal represented by somatosensory cortical input to the striatum should be reduced by 30% during eye blinks after the OO-weakening lesion, and the frequency of the blinks should be increased relative to the mean frequency of naturally occurring blinks. These two modifications should be done independently to evaluate the model's separate sensitivity to each.

### Model outputs

In terms of output, given the sequential chain of SNr influence on the trigeminal blink reflex circuit mediating both the TRBE and LCS, the model could use SNr output as a proxy for the excitability of the late (R2 and later) components of trigeminal blink system. In particular one could assay how SNr output changes after the first (“conditioning”) stimulus as compared to steady-state output patterns. Different scenarios based on the TRBE physiology in Schicatano et al. (1997) are outlined in Table 1.

### Incorporating clinical features

The model should also accommodate the stipulated role of the basal ganglia in mediating “state-to-motor” mappings and how they account for the “state-dependent” sensory tricks (“geste antagoniste”) known to ameliorate symptoms in some BEB patients (Ragothaman et al., 2007): if certain parts of the “state” space map to symptoms, the sensory trick can put the patient in a different part of the “state” space that does not map to motor symptoms. Similarly, BEB symptom expression may be dependent upon the “motor plan” aspect of “state,” as implied by cases where symptoms are induced or ameliorated by speech (Martino et al., 2010). With these design constraints, the model would unify the clinical observations of sensory tricks and task-dependencies with the suggested role of abnormal state-to-motor map learning in the development of BEB. Finally, the model should also be designed to be able to incorporate more precise characteristics of the patient phenotype as they become available. As an example, our recent efforts to leverage computer vision and machine learning technology to analyze symptoms of BEB from patient video recordings (Peterson et al., 2016) could be extended to examine the temporal patterns of various aspects of periocular motor control abnormalities, which would in turn serve as spatiotemporal constraints on the motor outputs of the blink and blink-related circuitry.

### Model testing/predictions

The model's predictions for TRBE can be measured in transgenic mouse models and the prediction of temporal dynamics in the basal ganglia output can be recorded with *in vivo* electrophysiological recordings in SNr. The transgenics should take advantage of advances in dystonia genetics, including the DYT1 gene and the more recently identified DYT25 gene (GNAL; which codes for a subunit of the G-protein mediating D1-type dopamine receptor signaling in the striatum). The model will also provide a platform for mechanistically integrating data about other potential factors in the pathophysiology of BEB, including for example how the roles of adenosine, acetylcholine, and endocannabinoids in striatal synaptic plasticity could influence network plasticity in the greater basal ganglia system. The combination of computer model simulations and animal model experiments would provide a rationale basis for further research into potential drug targets for patients.

## Broader significance

The framework and hypothesis put forth here link the specific patterns of adaptive “use” of the blink sensorimotor system induced by ophthalmic challenges such as dry eye to the repetitive sensorimotor learning functions of the basal ganglia. By prioritizing model features required to reproduce Schicatano's experimental findings, we can begin to develop an understanding of the circuit mechanisms by which ophthalmic and dopaminergic factors interact in BEB pathogenesis. Furthermore, the framework's predictions in the case of impairments to the striatal DA system provide hypotheses that can subsequently be experimentally tested in animal models with a variety of different protocols. As such, it is a step toward integrating the largely separate bodies of past research implicating local brainstem circuits and the basal ganglia in BEB. There are currently no strategies for preventing or curing BEB, and there are no treatment options that are completely efficacious. If we understood the pathogenesis of primary BEB we could develop principled strategies for preventing and reversing the disease. To the extent that a pathological sensorimotor learning process is part of the pathogenesis of BEB, this line of research could help get at the classic question of which aspects of pathophysiology reflect primary versus secondary, compensatory processes in dystonia, a matter prioritized in a recent update on BEB research (Valls-Sole and Defazio, 2016).

If BEB develops due to the joint interaction of ophthalmic and dopaminergic factors, the presence of either factor alone could be a detectable susceptibility indicating amelioration of that factor or proactive mitigation of the other factor to prevent the onset of BEB. Unfortunately, among the focal dystonias, the risk of spread in symptoms is among the highest in BEB (Weiss et al., 2006; Abbruzzese et al., 2008). Thus the same strategies used in preventing BEB onset in clinically normal but susceptible individuals may also be applicable for minimizing the risk of spread in existing patients with isolated BEB. Furthermore, if the model accounts for *how* factors interact in the pathogenesis of BEB, perhaps future investigations with the model can be used to suggest how exogenous manipulations of those factors could be used to renormalize “state to motor” mappings (as for example with specific acutely and carefully coordinated combinations of dopaminergic manipulations and sensorimotor retraining loosely analogous to that used in the focal limb dystonias, Zeuner and Hallett, 2003; Zeuner et al., 2005). If so, the model could ultimately be used to simulate the interventions prior to clinical use, helping to minimize risks to patients and maximize the likelihood of producing a cure.

Importantly, the hypothesis and framework developed here for BEB may also have broader relevance for other forms of dystonia. The view of the ophthalmic-triggered adaptations as changes in “use,” although under minimal if any volitional influence, provides a conceptual link between BEB and the focal task-specific dystonias, such as the focal hand dystonias (writer's cramp and musician's dystonia). The superior colliculus, a key node in the hypothesized BEB circuit, also mediates basal ganglia influence over oral motor learning (Taha et al., 1982), and so the circuits stipulated in the present model for BEB may also be implicated in oromandibular and/or laryngeal dystonia. Dystonia patients without BEB also exhibit increased TRBE (Nakashima et al., 1990), suggesting that understanding the mechanisms producing an increased TRBE may help improve our understanding of the pathogenesis of other forms of dystonia. Indeed, some of the same circuit mechanisms may be involved in many different types of dystonia (Defazio et al., 2007; Obermann et al., 2008), including even the generalized, childhood form of dystonia associated with the DYT1 gene, given evidence for reduced D2-family DA receptor availability in non-manifesting DYT1 carriers (Asanuma et al., 2005) and dopamine system abnormalities in DYT1 transgenic mice (Zhao et al., 2008).

## Author contributions

DP and TS: formulated idea, developed hypothesis and conceptual framework, and outlined model design. DP: Collected and synthesized relevant experimental literature.

## Funding

This study was funded by the Dystonia Coalition (NS065701 and TR001456), from the Office of Rare Diseases Research at the National Center for Advancing Translational Sciences and the National Institute of Neurological Disorders and Stroke, the Bachmann-Strauss Dystonia & Parkinson Foundation, the Benign Essential Blepharospasm Research Foundation, UCSD's Kavli Institute for Brain and Mind, the National Institute of Mental Health (NIMH 5T32-MH020002), and the National Science Foundation [the Temporal Dynamics of Learning Center, a Science of Learning Center (SMA-1041755) and the program in Mind, Machines, Motor Control (EFRI-1137279)], and the Howard Hughes Medical Institute.

### Conflict of interest statement

The authors declare that the research was conducted in the absence of any commercial or financial relationships that could be construed as a potential conflict of interest.
